# Genetic subtypes of multiple sclerosis severity are uncoupled from inflammatory lesion burden

**DOI:** 10.1007/s00401-026-03062-x

**Published:** 2026-08-21

**Authors:** Gianmarco Abbadessa, Karim L. Kreft, Owain Howell, Jean Victor Carlos de Oliveira, Benjamin Cooze, Artemis Papadaki, Joic Majo Jacinto, Ildiko Farkas, Yeung-Yeung Leung, Simona Bonavita, Emma Clare Tallantyre, Neil P. Robertson, Ai Nagano, Mie Rizig, Roberta Magliozzi, David Owen, Richard Reynolds, Richard Nicholas

**Affiliations:** 1 Department of Advanced Medical and Surgical Sciences, University of Campania Luigi Vanvitelli, Naples, Italy; 2 Department of Brain Sciences, Imperial College London, London, UK; 3 Academic Neurology, Academic Unit of Mental Health and Clinical Neurosciences, School of Medicine, University of Nottingham, Nottingham, UK; 4 Department of Neurology, Nottingham University Hospitals NHS Trust, Nottingham, UK; 5 Institute for Life Sciences, Swansea University, Swansea, UK; 6 Centre for Inflammatory Disease Monash Health, School of Clinical Sciences at Monash Health, Faculty of Medicine, Nursing and Health Sciences, Monash University, Melbourne, Australia; 7 Institute of Psychological Medicine and Clinical Neuroscience, Cardiff University, Cardiff, Wales UK; 8 Department of Neurology, University Hospital of Wales, Cardiff, Wales UK; 9 Department of Neurosciences, Biomedicine and Movement Sciences, University of Verona, Verona, Italy; 10 Department of Neuromuscular Diseases, UCL Queen Square Institute of Neurology, London, UK

**Keywords:** Multiple sclerosis, Genetic, Human Leukocyte Antigen, Chronic active lesions, Neuropathology, Disability progression

## Abstract

Determinants of long-term disability in multiple sclerosis (MS) remain incompletely understood, with clinical course varying greatly among individuals. Recent integrative genetic models have identified genomic clusters associated with divergent disability trajectories. Here, we integrated clinical, genetic, and postmortem neuropathological data from 291 MS brain donors from the UK MS Society Tissue Bank to test whether genomic severity clusters converge on distinct tissue phenotypes. Consistent with prior stratification, donors in genomic cluster 2 had lower age-adjusted disease duration score (ADDS) than cluster 3 (*β* = − 0.195, *P* = 0.023) and a shorter interval from progression to death than cluster 1 (*β* = − 0.225, *P* = 0.049). However, this adverse clinical profile did not reflect greater active or chronic active lesion burden. Instead, chronic active broad-rim lesions were more frequent in cluster 1 than clusters 2 and 3 (37.3% vs 24.0% and 27.2%; both FDR = 0.043), whereas chronic inactive lesions were enriched in clusters 2 and 3 relative to cluster 1 (58.2% vs 70.0% and 73.8%; FDR = 0.154 and 0.030, respectively). This dissociation was strongly influenced by HLA genetic background: cluster 1 carried the highest HLA genetic burden, which was associated with greater non-lesional HLA-D immunoreactivity (rho = 0.48, FDR < 0.001), higher HLA-DRB1 (rho = 0.514, FDR < 0.001) and HLA-DRB5 (rho = 0.740, FDR < 0.001) mRNA expression, more active lesions (rho = 0.21, FDR = 0.007) and chronic active broad-rim lesions (rho = 0.19, FDR = 0.017), and fewer chronic inactive lesions (rho = − 0.26, FDR < 0.001), but not with clinical severity. Although chronic active lesion burden remained a strong tissue correlate of severity, adjustment for it strengthened the association between cluster 2 and worse outcome (ADDS: cluster 2 vs 1: *β* = − 0.216, Holm-adjusted *P* = 0.038; cluster 2 vs 3: *β* = − 0.246, Holm-adjusted *P* = 0.012; time from progression to death: cluster 2 vs 1: *β* = − 0.288, Holm-adjusted *P* = 0.049). Overall, these findings indicate that (i) HLA genetic background shapes the neuropathological representation of inflammatory lesions, and (ii) the adverse clinical profile of cluster 2 is not fully explained by HLA-D-defined chronic active lesion burden in the sampled postmortem tissue, suggesting that genomic severity clusters and chronic active lesion burden capture complementary aspects of MS progression.

**Supplementary Information:**

The online version contains supplementary material available at 10.1007/s00401-026-03062-x.

## Introduction

Multiple sclerosis (MS) is a chronic inflammatory, demyelinating, and neurodegenerative disease of the central nervous system characterized by marked interindividual heterogeneity in long-term disability accumulation [24]. Differences in lesion area, topography, and subtype, among other measurable features, partially associate with MS severity and age [6, 9, 17, 19, 21, 23]. However, the variability in both the clinical and pathological extent is relatively poorly explained by standard demographic and clinical predictors [18]. Defining determinants of disease severity therefore remains a central challenge in MS research and a prerequisite for precision medicine.

Genome-wide association studies (GWAS) have greatly advanced our understanding of MS susceptibility and have identified more than 230 risk variants, most of which implicate immune pathways [11]. However, these variants have shown limited ability to explain differences in long-term disability [12], implying that the biology of disease onset and the biology of disease progression are distinct. This concept has been reinforced by recent genome-wide analyses of MS severity [12], which identified a significant association at the *DYSF–ZNF638* locus and showed heritability enrichment in central nervous system tissues, pointing to mechanisms of tissue resilience, and reserve as key determinants of long-term outcome. At the same time, the modest effect of individual loci indicates that progression heterogeneity is unlikely to be explained by a single variant [16].

These observations raise the possibility that clinically meaningful genetic architecture may emerge not at the level of individual alleles but through the combined action of susceptibility- and progression-related variants. Consistent with this view, integrative genetic models have recently identified genomic clusters associated with divergent disability trajectories [15]. Whether such genetically defined subgroups also map onto distinct neuropathological patterns remains unclear. Here, we assessed in a large cohort of MS brain donors from the national collection of the UK MS Society Tissue Bank, whether (i) measures of clinical disease trajectories were directionally concordant with prior prognostic stratification, and (ii) whether the genomic clusters differed in postmortem neuropathology. We show that genomic clusters associated with MS severity map onto distinct clinical and neuropathological patterns that are strongly shaped by Human Leukocyte Antigen (HLA) genetic background, while genomic clustering and chronic active lesion burden capture different aspects of disease progression.

## Materials and methods

We studied 291 UK Brain Bank MS donors with genome-wide genotyping and linked clinicopathologic data (Table 1). Prior to the start of the current study, we developed a predefined data-analysis plan (UK MS Society Tissue Bank registered study protocol n. 2,411,016), which was followed throughout the current study. Donors were assigned to one of three previously defined genomic clusters using genotype-derived weighted genetic risk scores (wGRS), mirroring the approach of the original study [15].

**Table 1:** Demographic, clinical, genetic, and neuropathological characteristics of the UK MS Brain Bank cohort overall and by genomic cluster

Variable	Overall	Cluster_1	Cluster_2	Cluster_3
Subjects, *n*	291	135	52	104
Female sex, *n*/*N* (%)	196/291 (67.4%)	93/135 (68.9%)	35/52 (67.3%)	68/104 (65.4%)
ADDS, median [IQR]	7.00 [4.00–9.00]	7.00 [5.00–9.00]	6.00 [3.75–9.00]	7.00 [4.00–10.00]
Age of onset, years, median [IQR]	31.00 [24.00–37.00]	32.00 [25.00–38.00]	30.50 [25.75–36.25]	28.00 [23.00–36.25]
Age at death, years, median [IQR]	61.00 [52.50–71.00]	62.00 [53.50–71.00]	62.00 [48.75–71.00]	61.00 [52.00–69.50]
Time to death (disease duration), years, median [IQR]	29.00 [22.00–37.50]	30.00 [21.50–37.00]	27.50 [17.75–36.25]	30.00 [23.75–38.25]
Time to progression, years, median [IQR]	10.00 [3.00–18.00]	10.00 [3.00–17.00]	9.50 [2.00–19.75]	11.00 [4.00–20.50]
Time from progression to death, years, median [IQR]	16.00 [11.00–22.00]	16.00 [11.00–22.75]	15.00 [8.00–18.75]	17.00 [11.50–24.00]
HLA-DRB1*15:01 positive, *n*/*N* (%)	154/284 (54.2%)	86/132 (65.2%)	20/49 (40.8%)	48/103 (46.6%)
HLAGB, median [IQR]	0.89 [0.18–1.19]	1.00 [0.58–1.30]	0.89 [0.07–1.11]	0.61 [0.13–1.14]
HLA-D immunoreactivity, %, median [IQR]	3.16 [1.71–5.04]	3.40 [2.18–5.44]	2.58 [1.44–4.18]	3.20 [1.37–4.71]
Active lesion present, *n*/*N* (%)	63/287 (22.0%)	31/134 (23.1%)	9/50 (18.0%)	23/103 (22.3%)
Active lesion burden, median [IQR]	0.00 [0.00–0.00]	0.00 [0.00–0.00]	0.00 [0.00–0.00]	0.00 [0.00–0.00]
Chronic active BRL − lesion present, *n*/*N* (%)	192/287 (66.9%)	93/134 (69.4%)	29/50 (58.0%)	70/103 (68.0%)
Chronic active BRL − lesion burden, median [IQR]	2.00 [0.00–4.00]	2.00 [0.00–4.00]	1.00 [0.00–3.00]	2.00 [0.00–4.00]
Chronic active BRL + lesion present, *n*/*N* (%)	90/287 (31.4%)	50/134 (37.3%)	12/50 (24.0%)	28/103 (27.2%)
Chronic active BRL + lesion burden, median [IQR]	0.00 [0.00–1.00]	0.00 [0.00–1.00]	0.00 [0.00–0.00]	0.00 [0.00–1.00]
Chronic inactive lesion present, *n*/*N* (%)	189/287 (65.9%)	78/134 (58.2%)	35/50 (70.0%)	76/103 (73.8%)
Chronic inactive lesion burden, median [IQR]	1.00 [0.00–2.00]	1.00 [0.00–2.00]	1.00 [0.00–2.00]	1.00 [0.00–2.00]
Total lesion burden, median [IQR]	4.00 [1.00–8.00]	4.50 [1.25–8.00]	3.00 [1.00–5.00]	4.00 [1.00–7.50]

Values are presented as *n*/*N* (%) for categorical variables and median [IQR] for continuous variables. Genomic clusters were assigned using the published clustering framework applied to genotype-derived weighted genetic risk profiles. Lesion presence indicates the proportion of donors with at least one lesion of the specified subtype, whereas lesion burden refers to the aggregated lesion count across evaluable regions. Sample sizes for median-based variables were: ADDS, age of onset, age at death, disease duration, and HLAGB: overall *n* = 291, Cluster_1 *n* = 135, Cluster_2 *n* = 52, Cluster_3 *n* = 104; time to progression and time from progression to death: overall *n* = 253, Cluster_1 *n* = 118, Cluster_2 *n* = 44, Cluster_3 *n* = 91; HLA-D immunoreactivity: overall *n* = 269, Cluster_1 *n* = 128, Cluster_2 *n* = 49, Cluster_3 *n* = 92; lesion burden variables: overall *n* = 287, Cluster_1 *n* = 134, Cluster_2 *n* = 50, Cluster_3 *n* = 103. ADDS, age-adjusted disease duration score; HLAGB, HLA genetic burden; BRL, broad-rim lesion

### Ethics approval

Postmortem brain tissue from MS donors was obtained via the UK MS Society Tissue Bank at Imperial College London. Tissue collection and autopsy were performed with prior informed consent and in accordance with approval from the National Research Ethics Committee (08/MRE09/31). The study was conducted in compliance with the Code of Ethics of the World Medical Association (Declaration of Helsinki).

### Clinical and neuropathological outcomes

The MS Society Tissue Bank collects postmortem tissue from MS and non-MS control donors nationwide, together with death certificates and clinical records. Clinical notes were used to determine the age at symptom onset, onset of the progressive phase, defined as gradual disability worsening for at least 1 year without relapses [22], and death. Clinical outcomes were age-adjusted disease duration score (ADDS), time from symptom onset to progression (time to progression), time from progression to death. ADDS is a composite score (1–13) obtained by summing an age at death score (ADS; 0–6) and a disease duration score (DDS; 1–7). ADS and DDS increase with older age at death and longer disease duration, respectively, and were assigned using cohort mean/standard deviation (SD)-based brackets. Higher ADDS reflects milder disease course.

Neuropathological assessments were performed on six systematically sampled brain blocks (selected by the attendant neuropathologist) which comprised 1) superior frontal gyrus grey and white matter, 2) cingulate gyrus grey and white matter, including the angle of the lateral ventricle, 3) occipital lobe grey and white matter, including tissue adjacent to the occipital horn of the lateral ventricle, 4) thalamus (sampled at the level of the mammillary bodies), 5) basal pons, and 6) cerebellar white matter and dentate nucleus. These regions represent both cortical and non-cortical sites frequently affected in MS [7].

Immunostaining and tissue characterization: Microglia/macrophage density was captured by HLA-D immunoreactivity using a mouse anti-HLA-DP/DQ/DR antibody (clone CR3/43 [14]; Dako, Glostrup, Denmark), and myelin integrity with anti-myelin oligodendrocyte glycoprotein antibody (MOG; clone z12, in house). Detection and visualization was performed using an HRP-conjugated anti-mouse secondary antibody (ImmPRESS HRP, Vector Laboratories, UK) with diaminobenzidine (ImmPACT DAB, Vector Labs, UK). All sections were counterstained with hematoxylin, DePeX mounted and digitized at 40-200X using an Aperio AT2 slide scanner (Leica Bio systems, Nussloch, Germany).

Capturing neuropathological outcomes: Diffuse microglia/macrophage activation was measured using HLA-D immunoreactivity in non-lesional areas of superior frontal gyrus grey and white matter, thalamus and basal pons (donors *n* = 269, blocks *n* = 1,480). Non-lesion areas were areas of normal-appearing matter ≥ 10 mm from the nearest visible demyelinated lesion [1, 8, 17]. The percent HLA-D-positive area was quantified from two annotations (5–8 mm^2^ per annotation) using the positive-pixel classifier tool in QuPath (version 0.5) [2, 3]. The presence and number of active, chronic active broad-rim lesion positive (BRL + , defined as a chronic active lesion with a HLA-D + rim ≥ 1 mm; [14]), chronic active broad-rim lesion negative (chronic active BRL −), or chronic inactive lesions [19] were captured from all non-cortical grey matter regions per block per donor.

For each donor, lesion counts were aggregated across sampled regions according to four categories: active (A), chronic active BRL − , chronic active BRL + (referred to as BRL +), and chronic inactive (CI) (donors *n* = 287, blocks *n* = 1,545). Because the number of assessed regions varied by donor and lesion type, lesion count was expressed per number of evaluable regions, to account for differential sampling.

### Genotyping, quality control, and imputation

Genotyping was performed using the Illumina Infinium Omni 2.5 Exome-8 BeadChip array, which assessed 2,619,927 variants, including repeated markers, covering tag SNPs from the HapMap and 1000 Genomes projects. Quality control was carried out with PLINK v1.90b6.21 by excluding individuals with sex discrepancies, genotyping call rates below 95%, or heterozygosity rates with |F|> 0.1, and by removing variants with minor allele frequency below 0.05 or call rate below 0.9. After sample QC, 300 MS individuals and 1,295,383 variants remained. Genotypes were then harmonized and imputed against the 1000 Genomes Phase III reference panel using SHAPEIT2 [4] and IMPUTE2 [10], retaining SNPs with MAF ≥ 0.05, completion rate ≥ 0.9, and imputation information score ≥ 0.8, resulting in 6,432,503 variants after QC.

### Genomic clustering analysis

To replicate the genomic clustering strategy described by Kreft et al. [15], the SNP dataset was used to calculate genomic risk scores based on MS susceptibility and progression variants reported in previous GWAS [11–13], including the HLA genomic burden score, with each variant assigned to a single score. Two variants were not available for the current analysis: rs11809700 and rs17780048. No variants in high-linkage disequilibrium were available, and therefore these two variants were excluded from the current analysis. Residual missing genotype calls among score variants were rare (Fig. S1) and were replaced using the expected allele dosage (2 × effect minor allele frequency). For each individual, risk alleles were counted and weighted according to their reported effect sizes, generating individual genomic risk profiles. K-nearest neighbor algorithm was applied to assign genomic clusters based on the original Welsh clustering [15]. Population structure was evaluated using principal component analysis derived from the genotype data, in order to assess whether cluster assignment was independent of ancestry-related variation. See Ref. [15] for details. Figure S2 shows the cumulative genetic burden for each score in our population compared to those of the Welsh MS cohort [15]. The SNPs included in the clustering framework and descriptive summaries of the genetic scores across clusters are provided in Supplementary Tables 1 and 2, respectively.

### RNA sequencing

RNA was extracted from 226 pons tissue blocks using the Qiagen RNeasy Lipid Tissue Mini Kit. Libraries were prepared with the Lexogen QuantSeq 3′ mRNA FWD kit and sequenced on the NextSeq 2000. Reads were processed with the nf-core/rnaseq pipeline (v3.3) in Nextflow (v21.04.0), aligned to the reference genome with STAR, sorted with samtools, and quantified at gene level with RSEM to obtain read counts and transcript per million (TPM) values. PCA-based quality control of the RNA-seq dataset identified 28 outlier samples, defined as 23 extreme outliers outside the 99% Mahalanobis-distance threshold and 5 additional borderline outliers between the 90% and 99% thresholds, and these samples were excluded (Fig. S3). Of the remaining 198 samples, 8 could not be matched to the genetic dataset required for downstream analyses, leaving 190 samples for the final expression analyses. For the present study, TPM values for only HLA-DRB1 and HLA-DRB5 were extracted from the full expression matrix.

### Statistical analysis

Differences in clinical severity outcomes (ADDS, time to progression, and time from progression to death) were assessed using linear regression models fitted to log-transformed outcomes and adjusted for sex and age at onset. Pairwise comparisons were performed for cluster 1 versus cluster 2, cluster 1 versus cluster 3, and cluster 2 versus cluster 3, and regression coefficients, 95% confidence intervals (CIs), and *P* values were reported.

For neuropathological analyses, lesion presence (proportion of cases with at least one lesion) and lesion count were first examined descriptively across clusters for each lesion subtype. Nominal overall three-group tests were reported using contingency tests for lesion presence and Kruskal–Wallis tests for lesion burden/rate. To further explore whether simple pairwise differences in lesion presence supported the descriptive patterns, logistic regression models were fitted (cluster 1 versus cluster 2 and cluster 1 versus cluster 3), adjusted for sex and age at death. *P* values from these pairwise logistic comparisons were adjusted using the Benjamini–Hochberg false discovery rate (FDR) method within each lesion subtype.

HLA genetic burden was compared across clusters using linear regression adjusted for sex. HLA-D immunoreactivity was compared across clusters using linear regression adjusted for age at death and sex, including a contrast comparing cluster 1 versus clusters 2 and 3 combined. For HLA-related tissue analyses, pontine HLA-DRB1 and HLA-DRB5 expression were compared across clusters using linear regression models adjusted for tissue RNA quality (RNA integrity score; RIN), age at death, and sex. Pairwise contrasts were corrected within outcome using the Benjamini–Hochberg FDR method. Associations between HLA genetic burden and HLA-D immunoreactivity or HLA-DRB1 and HLA-DRB5 expression were assessed using Spearman correlation in the overall cohort and within each genomic cluster. *P* values were corrected for multiple testing using the Benjamini–Hochberg FDR method in the overall cohort and separately within each genomic cluster.

Associations between HLA-DRB1*15:01 positivity and clinical severity were assessed using linear regression models fitted to log-transformed clinical outcomes and adjusted for sex and age at onset. Associations between lesion burden or non-lesional HLA-D immunoreactivity and clinical severity were assessed using linear regression models on log-transformed clinical outcomes, adjusted for sex and age at onset, in the overall cohort and separately within each genomic cluster. Neuropathologic measures were entered as continuous predictors, and effect estimates were reported as regression coefficients per 1 SD increase. *P* values were corrected for multiple testing using the Benjamini–Hochberg FDR method in the overall cohort and separately within each genomic cluster.

Finally, to assess whether HLA-related measures or chronic active lesion burden, defined as the combined burden of chronic active BRL − and BRL + lesions, modified the association between genomic cluster assignment and clinical severity, linear regression models on log-transformed clinical outcomes were fitted with adjustment for sex and age at onset. Additional models further adjusted for HLA-DRB1*15:01 status, HLA genetic burden, chronic active lesion burden, and combinations of these covariates. Analyses were performed for the pairwise comparisons of cluster 1 versus cluster 2, cluster 1 versus cluster 3, and cluster 2 versus cluster 3. Regression coefficients, 95% CIs, and *p* values were reported, with Holm adjustment applied within each outcome-comparison block across the multivariable models. Analyses were performed in R (Version 4.4.1).

## Results

### Clinical replication of genomic clusters in the UK MS brain bank

**Fig. 1:**
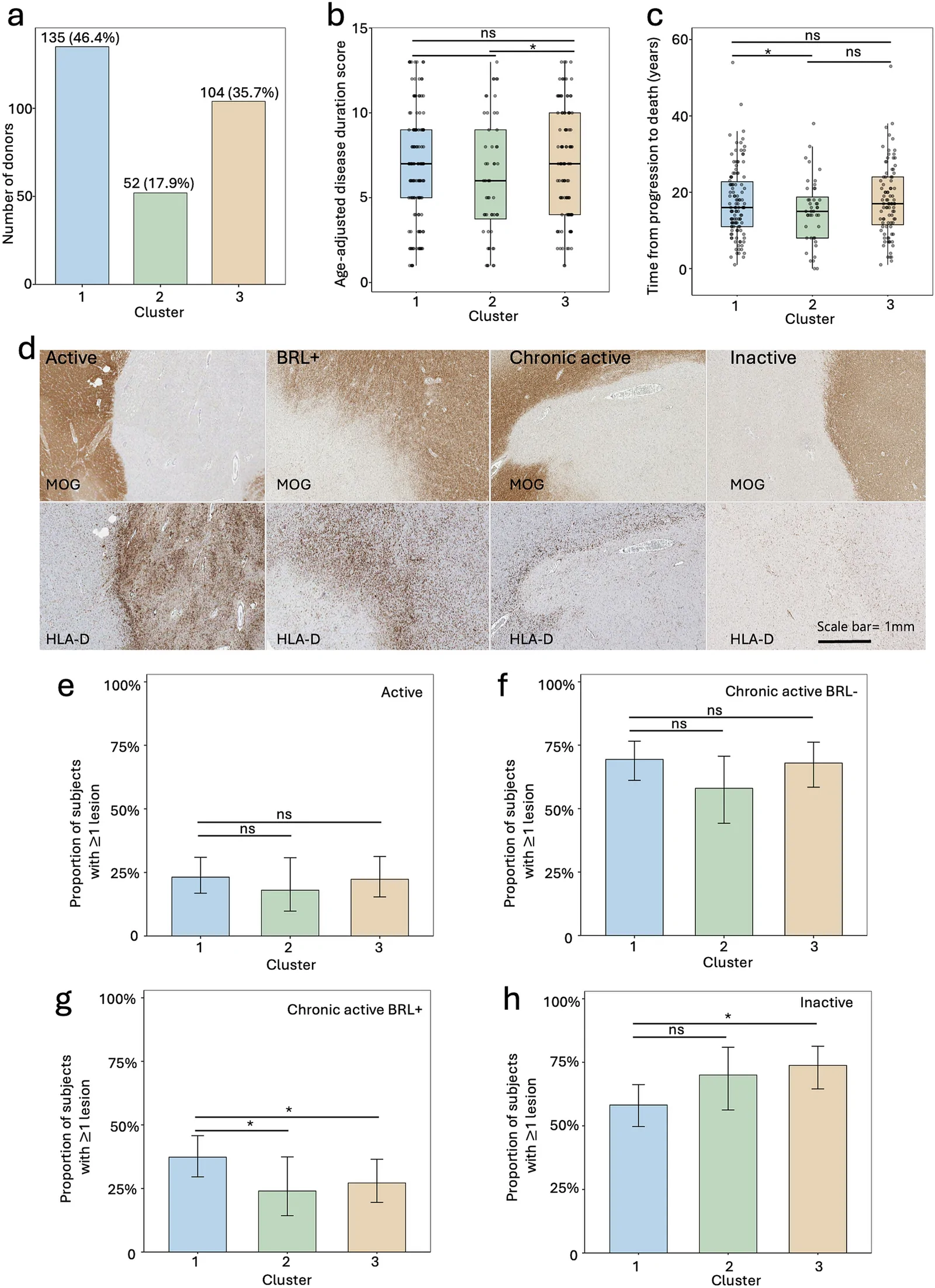
Clinical replication of genomic clusters and associated lesion profile in the UK Tissue Bank cohort. **a** Number of donors assigned to each genomic cluster in the UK MS Brain Bank cohort. **b** Age-adjusted disease duration score (ADDS) across genomic clusters. **c** Time from progression to death across genomic clusters. Differences in ADDS and time from progression to death between clusters were assessed using linear regression models adjusted for sex and age at onset. For panels (**b**) and (**c**), * indicates *P* value < 0.05. **d** Lesions (myelin oligodendrocyte glycoprotein—MOG) of the white and non-cortical grey matter were classified as active, chronic active with a broad rim (BRL +), chronic active (without a broad rim) or chronic inactive, based on the density and distribution of HLA-D + microglia/macrophages. **e**–**h** Descriptive proportion of donors with at least one lesion of each subtype across genomic clusters: active lesions (**e**), chronic active BRL-negative lesions (**f**), chronic active BRL-positive lesions (**g**), and chronic inactive lesions (**h**). Differences in lesion presence across clusters were assessed using logistic regression models for cluster 1 versus cluster 2 and cluster 1 versus cluster 3, adjusted for sex and age at death. Error bars indicate 95% confidence intervals for the observed proportions. For panels (**e**–**h**), * indicates FDR < 0.05

We first determined whether applying the published genomic clustering framework [15] to our UK MS brain bank cohort would reproduce the expected direction of clinical severity. In donors with both clinical and genomic cluster data (*n* = 291), 135 were assigned to cluster 1, 52 to cluster 2, and 104 to cluster 3 (Fig. 1a), and the proportion of individuals within each cluster was similar to the original study [15]. Consistent with the original prognostic stratification, cluster 2 identified a subgroup with a more adverse disease course than the other clusters. Donors in cluster 2 had the shortest time to death, with a median of 27.5 years compared with 30.0 years in both clusters 1 and 3 (Table 1); this translates into a lower ADDS in cluster 2 than in clusters 1 and 3 (median 6.0 versus 7.0 and 7.0), with a significant difference between clusters 2 and 3 (*β* =  − 0.195, 95% CI − 0.362 to − 0.027, *P* = 0.023; Fig. 1b and Supplementary Table 3). In addition, cluster 2 showed the shortest interval from progression to death (Table 1); this was significantly shorter than cluster 1 (*β* =  − 0.225, 95% CI − 0.448 to − 0.001, *P* = 0.049; Fig. 1c and Supplementary Table 3) and showed a similar trend versus cluster 3 (*β* =  − 0.214, 95% CI − 0.460 to 0.032, *P* = 0.088; Fig. 1c and Supplementary Table 3). Time to progression was comparable across clusters (Supplementary Table 3), suggesting that the poorer prognosis associated with cluster 2 was not driven by earlier conversion to progressive disease, but rather by shorter overall time to death and reduced survival after progression.

Treatment exposure was unlikely to explain these differences: medication data were available for 148 of 291 donors, and as expected from a postmortem cohort, most had no documented disease-modifying therapy exposure (135/148, 91.2%), and treatment distribution did not differ among clusters. Together, these findings support cluster 2 as the clinically most severe genomic subgroup, in line with the published prognostic stratification [15].

### Cluster-associated lesion patterns point to an HLA-linked tissue signal

We then addressed whether genomic clusters have a neuropathologic counterpart. In descriptive analyses of lesion presence (≥ 1 lesion; *n* donors = 287), cluster 1 showed the highest frequency of chronic active BRL + lesions, whereas clusters 2 and 3 showed higher frequencies of chronic inactive lesions (Fig. 1d–h and Supplementary Table 4). In logistic regression models adjusted for sex and age at death, cluster 2 and cluster 3 were both less likely than cluster 1 to harbor chronic active BRL + lesions (FDR = 0.043 for both comparisons; Fig. 1g), while cluster 3 was more likely than cluster 1 to harbor chronic inactive lesions (FDR = 0.030; Fig. 1h); the corresponding contrast for cluster 2 was in the same direction but did not reach significance. No between-cluster differences were detected with regard to active, chronic active BRL-negative (Fig. 1e–f and Supplementary Table 4). Analysis of lesion counts did not show clear overall between-cluster differences, although the directional pattern for chronic inactive lesions and chronic active BRL + counts was concordant with the lesion presence analysis (Supplementary Table 4). Together, these findings suggest that the cluster signal was driven primarily by lesion phenotype, particularly the relative enrichment of chronic active BRL + pathology in cluster 1 and chronic inactive pathology in clusters 2 and 3, rather than by overall lesion burden. Thus, the cluster with the more rapidly progressive disease course (cluster 2) was, somewhat counterintuitively, less likely to have detectable chronic active BRL + at end stage.

**Fig. 2:**
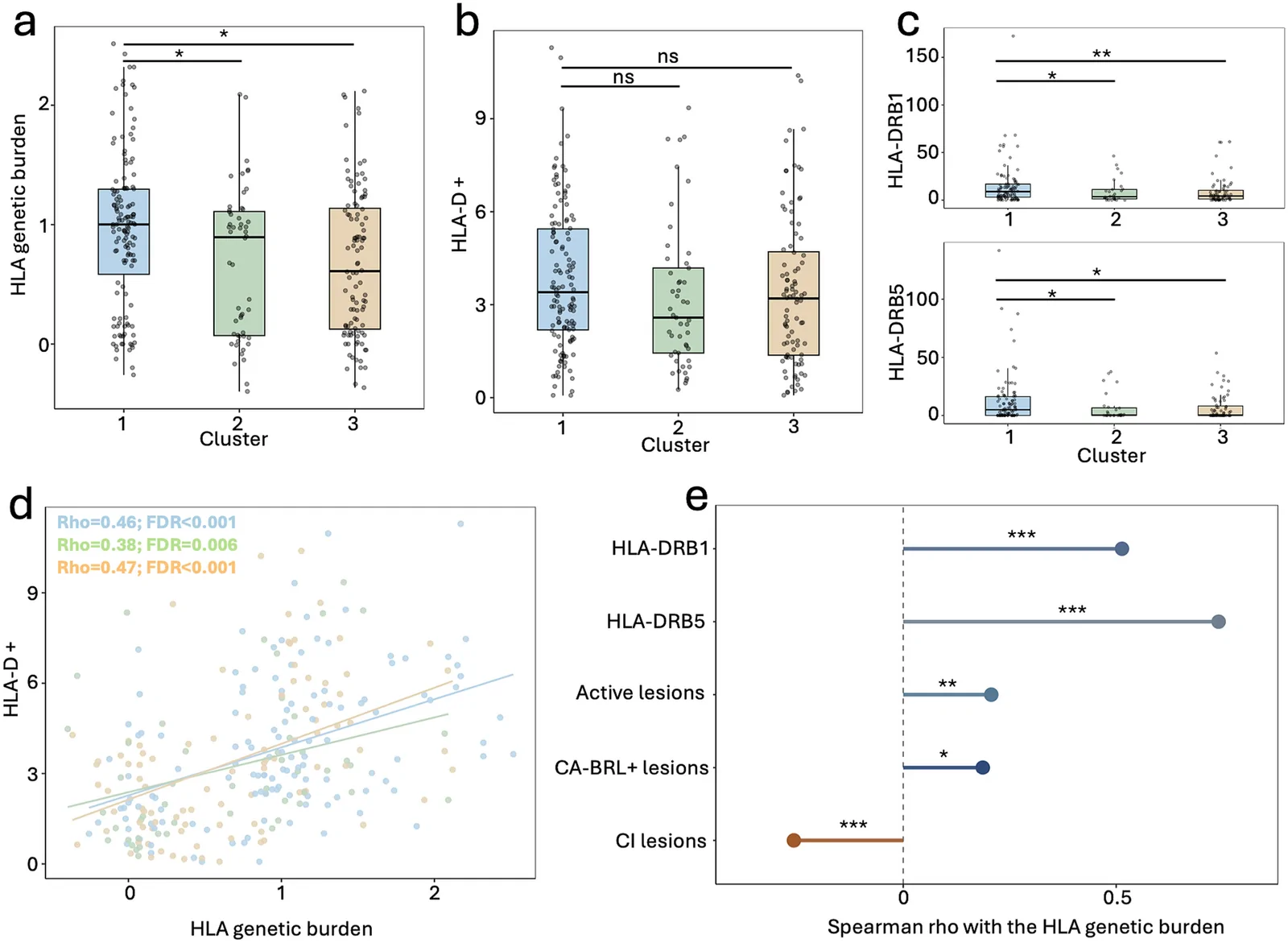
The HLA axis links genetic burden, tissue HLA signal, and lesion phenotype. **a** HLA genetic burden (HLAGB) across genomic clusters; cluster differences were assessed using linear regression models adjusted for sex. **b** Non-lesional HLA-D immunoreactivity across genomic clusters; cluster differences were assessed using linear regression models adjusted for sex and age at death. **c** Pontine HLA gene expression across genomic clusters, shown separately for HLA-DRB1 and HLA-DRB5; cluster differences were assessed using linear regression models adjusted for tissue RNA integrity score, age at death, and sex. **d** Association between HLA genetic burden and non-lesional HLA-D immunoreactivity at the individual level. Points and fitted lines are colored by genomic cluster. **e** Summary of the associations between HLA genetic burden and HLA-related tissue and lesion measures, including HLA-DRB1 expression, HLA-DRB5 expression, active lesions, chronic active BRL-positive lesions, and chronic inactive lesions. Associations were assessed using Spearman correlation. Significance is reported using FDR-adjusted values: * indicates FDR < 0.05, ** indicates FDR < 0.01, and *** indicates FDR < 0.001

These lesion patterns, considered alongside the clinical findings, raised an interpretive question as to what biological dimension the genomic clusters represent in tissue. This was particularly relevant because lesion phenotype is typically defined using HLA-D immunostaining [14, 19], and the clustering framework is partly based on an HLA genetic burden score, which was higher in cluster 1 than in clusters 2 and 3 (Fig. 2a). We therefore tested whether the HLA genetic component was associated with a measurable imprint in non-lesional tissue.

In line with the differences observed in HLA genetic burden across the three clusters (Fig. 2a), HLA-D immunoreactivity (a marker of diffuse microglia/macrophage activation, *n* = 269) was higher in cluster 1 than in clusters 2 and 3 (*n* = 141; median [IQR] %: cluster 1, 3.403 [2.185–5.443]; clusters 2 + 3, 3.072 [1.382–4.482]; *P* = 0.027). The pairwise contrasts showed the same direction of effect, but neither cluster 1 versus cluster 2 (*P* = 0.047; BH-adjusted *P* = 0.071) nor cluster 1 versus cluster 3 (*P* = 0.071; BH-adjusted *P* = 0.071) remained significant after multiple-testing correction (Fig. 2b). Across all donors with paired measures (*n* = 269), HLA genetic burden showed a strong positive correlation with HLA-D immunoreactivity (Spearman *ρ* = 0.475; *P* < 0.001; FDR < 0.001; Fig. 2d). Importantly, the correlation persisted within each cluster: cluster 1 (*n* = 128; *ρ* = 0.464; FDR < 0.001), cluster 2 (*n* = 49; *ρ* = 0.388; *P* = 0.006; FDR = 0.006), and cluster 3 (*n* = 92; *ρ* = 0.471; *P* < 0.001; FDR < 0.001; Fig. 2d), supporting an individual-level link between HLA genetics and tissue HLA-D + staining beyond cluster membership alone.

### A graded HLA axis connects genetic burden, non-lesional HLA gene expression, and HLA immunoreactivity

To further probe this HLA-dominant non-lesional tissue signal, we examined whether HLA genetic burden was mirrored at the transcript level in MS-relevant HLA genes. In patients with bulk RNA expression data (*n* = 190), HLA-DRB1 expression was lower in clusters 2 and 3 than in cluster 1 (median [IQR]: cluster 2, 3.560 [1.268–11.258] vs cluster 1, 8.950 [3.070–16.870], FDR = 0.041; cluster 3, 4.300 [1.130–10.210] vs cluster 1, 8.950 [3.070–16.870], FDR = 0.005; Fig. 2c). HLA-DRB5 expression showed the same pattern (median [IQR]: cluster 2, 0.270 [0.000–6.547] vs cluster 1, 4.930 [0.000–16.255], FDR = 0.028; cluster 3, 0.210 [0.000–8.225] vs cluster 1, 4.930 [0.000–16.255], FDR = 0.028) (Fig. 2c). Across individuals, mRNA expression strongly correlated with the HLA genetic burden score (HLA-DRB1 *ρ* = 0.514, *P* < 0.001, FDR < 0.001; HLA-DRB5 *ρ* = 0.740, *P* < 0.001, FDR < 0.001; Fig. 2e), with consistent within-cluster correlations (HLA-DRB1: cluster 1 *ρ* = 0.434, FDR < 0.001; cluster 2 *ρ* = 0.688, FDR < 0.001; cluster 3 *ρ* = 0.442, FDR < 0.001; HLA-DRB5: cluster 1 *ρ* = 0.691, FDR < 0.001; cluster 2 *ρ* = 0.743, FDR < 0.001; cluster 3 *ρ* = 0.762, FDR < 0.001). Together, these results indicate that a graded HLA signature, spanning genetic burden, HLA-DRB expression, and non-lesional HLA-D immunoreactivity, emerged as a dominant non-lesional tissue-level correlate.

### HLA genetic burden is associated with lesion subtype burden but not with clinical severity

To further define the relationship between HLA genetics, lesion subtype, and clinical severity, we tested whether HLA genetic burden was associated with lesion subtype distribution independently of genomic cluster assignment. Higher HLA genetic burden was associated with fewer chronic inactive lesions (CI: Spearman *ρ* =  − 0.26, *P* < 0.001, FDR < 0.001; Fig. 2e and Supplementary Table 5), and with more active lesions (A: *ρ* = 0.21, *P* < 0.001, FDR = 0.007; Fig. 2e and Supplementary Table 5) and chronic active BRL + (*ρ* = 0.19, *P* < 0.001, FDR = 0.017; Fig. 2e and Supplementary Table 5). These findings indicate that higher HLA genetic burden is associated with a more inflammatory lesion profile and further support the interpretation that HLA-related genetics is a determinant of lesion subtype distribution when assessed by HLA-D immunostaining. By contrast, HLA-DRB1*15:01 positivity was not associated with ADDS (*P* = 0.677) or with other clinical outcomes (Supplementary Table 6). Together, these findings indicate that HLA-related genetics is closely linked to inflammatory lesion subtype, when assessed by anti-HLA-D immunochemistry, but not to disease severity. This raised the possibility that HLA-related genetics may influence lesion phenotype without directly determining clinical severity, thereby contributing to the discordance between cluster-associated clinical severity and postmortem chronic active lesion burden.

### Chronic active lesion burden is a severity marker while non-lesional HLA immunoreactivity is not

Because cluster 2 showed greater clinical severity despite a lower probability of chronic active BRL + lesions at death, we next asked whether lesion burden was associated with severity within each genomic cluster. Consistent with established neuropathologic markers of progression, higher lesion burden was associated with greater clinical severity in the overall cohort and, importantly, within each genomic cluster. Detailed results are provided in Supplementary Table 7. The strongest and most consistent associations were observed for ADDS, for which total lesion burden and chronic active lesion burden, including both BRL-negative and BRL-positive lesions, were significantly associated with worse severity in the overall cohort and across clusters. Similar but less consistent associations were observed for time to progression and time from progression to death: in the overall cohort, higher total lesion burden and higher chronic active BRL- and BRL + lesion burden were associated with shorter time to progression. Within clusters, these time-based associations were less robust after multiple-testing correction. BRL + lesion burden remained clearly associated with severity in clusters 1 and 3, but was weaker and less consistent in cluster 2, in keeping with the lower probability of BRL + pathology in this group. Active lesion burden showed a more limited pattern overall, whereas chronic inactive lesion burden showed no meaningful association with severity across outcomes or clusters. Non-lesional HLA-D immunoreactivity showed no consistent association with clinical severity in the whole cohort (Supplementary Table 7). Together, these findings indicate that the apparent discordance between cluster-level severity and lesion subtype does not reflect a failure of chronic active lesion burden as a marker of severity in this cohort. Rather, they suggest that the cluster 2 genetic profile captures aspects of disease severity that are not fully represented by end-stage active or chronic active lesion burden.

### Chronic active lesion burden modifies the association between genomic cluster assignment and clinical severity

**Fig. 3:**
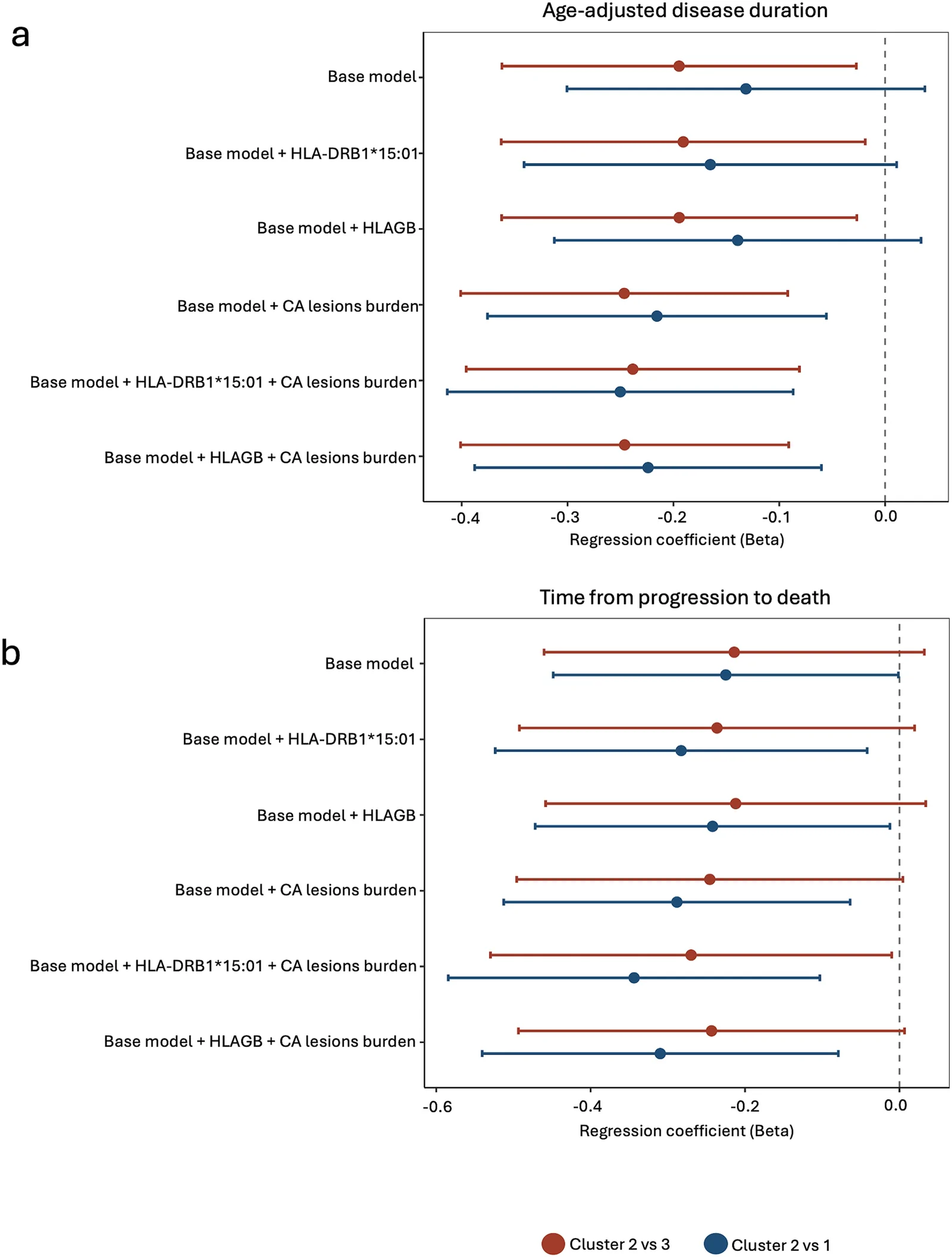
Chronic active lesion burden modifies the association between genomic cluster assignment and clinical severity. Forest plots show regression coefficients and 95% confidence intervals for ADDS (**a**) and time from progression to death (**b**), comparing cluster 2 versus cluster 1 and cluster 2 versus cluster 3 across sequentially adjusted multivariable linear regression models. Models included adjustment for sex and age at onset (base model), sex and age at onset plus HLA-DRB1*15:01, sex and age at onset plus HLAGB, sex and age at onset plus chronic active lesion burden, sex and age at onset plus chronic active lesion burden plus HLA-DRB1*15:01, and sex and age at onset plus chronic active lesion burden plus HLAGB. Points indicate beta coefficients and horizontal bars indicate 95% confidence intervals. Significance is reported in Table S8

Having established that lesion burden remained associated with severity within clusters, and that HLA-related measures shaped inflammatory lesion phenotype without directly mapping onto clinical severity, we next tested whether HLA genetic background or chronic active lesion burden modified the association between genomic cluster assignment and clinical outcome. Adjustment for HLA-DRB1*15:01 or HLA genetic burden alone did not consistently strengthen the association of cluster 2 with clinical outcomes (Fig. 3 and Supplementary Table 8). By contrast, after adjustment for chronic active lesion burden, defined as the combined burden of chronic active BRL − and BRL + lesions, the association between cluster 2 and worse outcome became stronger for both ADDS (cluster 2 vs 1: *β* =  − 0.216, 95% CI − 0.376 to − 0.055, Holm-adjusted *P* = 0.038; cluster 2 vs 3: *β* =  − 0.246, 95% CI − 0.401 to − 0.092, Holm-adjusted *P* = 0.012; Fig. 3a) and for time from progression to death (cluster 2 vs 1: *β* =  − 0.288, 95% CI − 0.513 to − 0.064, Holm-adjusted *P* = 0.049; Fig. 3b), whereas time to progression remained comparable between clusters (Supplementary Table 8). The observation that time to progression still remained comparable across clusters, even after adjustments, strengthens the hypothesis that the poorer prognosis associated with cluster 2 was not driven by earlier conversion to progressive disease, but rather by reduced survival after progression. Further adjustment for HLA-DRB1*15:01 or HLA genetic burden supported the same pattern, with the strongest effects observed in models including chronic active lesion burden together with HLA-DRB1*15:01 (Supplementary Table 8). Together, these findings indicate that chronic active lesion burden partly attenuates the observable clinical severity signal of cluster 2, whereas HLA-related measures do not account for this effect, strengthening the hypothesis that the cluster 2 genetic profile captures components of disease severity that are not fully represented by end-stage active and chronic active lesion burden.

## Discussion

Polygenic risk scores stratified MS cases into genetically defined groups with distinct disability trajectories. Defining the pathological features associated with these clusters may provide insight into the biological determinants of disease severity. In this independent UK MS brain bank cohort, the previously published genomic clustering framework reproduced the expected clinical pattern [15], with cluster 2 showing the least favorable course. However, the neuropathologic correlate was not a simple increase in active or chronic active lesion burden: despite its worse clinical profile, cluster 2 was less likely to show chronic active broad-rim lesions, even though these lesions are widely regarded as markers of aggressive MS pathology.

This apparent discordance led us to examine HLA-linked tissue biology, because cluster 2 carried a lower HLA genetic burden, whereas lesion activity states are commonly classified using HLA-based histopathology [14]. Since HLA-D expression may itself be influenced by HLA genetic background [25], HLA-related genetics could affect the histological representation of inflammatory lesion activity. Consistent with this possibility, we observed that non-lesional HLA-D immunoreactivity differed across clusters in a pattern consistent with HLA genetic burden, and that HLA genetic burden strongly correlated with both HLA-DRB1/HLA-DRB5 gene expression and HLA-D protein immunoreactivity, while also being associated with active and chronic active BRL + lesion burden, suggesting that HLA-linked immunogenetic variation may influence HLA-D-defined lesion phenotype and its histological classification rather than clinical severity itself. Importantly, this does not diminish the relevance of chronic active lesions as markers of aggressive disease. Chronic active lesion burden remained associated with worse clinical outcome both in the cohort overall and within clusters. Therefore, we tested whether the cluster 2 genetic profile predicted MS severity independently of chronic active lesion burden, an interpretation supported by regression models in which adjustment for chronic active lesion burden substantially strengthened the association between cluster 2 and worse outcome. Taken together, these findings indicate that genomic clusters and chronic active lesions are both prognostically relevant, but capture partly non-overlapping dimensions of MS progression.

It is important to emphasize that this apparent discordance should not be interpreted as contradicting previous studies. Klotz et al. [14] started from clinically defined rapidly versus slowly progressive MS and identified broad-rim lesions as markers of aggressive disease, a finding also supported by our cohort. Our study addressed a related but distinct question: whether genetically defined severity subgroups map onto the same pathological phenotype. In this framework, the clinically most severe genomic cluster was not characterized by a greater burden of HLA-D-defined broad-rim lesions.

This finding can be reconciled by considering the different relationships linking HLA biology, chronic active lesion classification, and clinical severity. HLA-axis biology was not associated with severity measures in this cohort, consistent with large-scale genetic studies showing that HLA-DRB1*15:01 is not related to long-term disease outcomes [12, 13, 16]. By contrast, lesion burden, particularly chronic active lesion burden, remained associated with a more rapidly progressive disease course across the cohort and within genomic clusters. Thus, our data preserve the expected lesion-severity relationship and support the established relevance of chronic active lesions as markers of severe disease.

At the same time, HLA genetic background may influence HLA gene expression and the degree of HLA-D immunoreactivity on innate immune cells, thereby affecting whether lesions meet HLA-D-based criteria for active, chronic active, or BRL + pathology. This introduces a potential circularity, whereby HLA genetic burden may increase the likelihood of detecting HLA-D-defined inflammatory lesion activity independently of the underlying inflammatory burden [25]. However, HLA-D-based classification alone is unlikely to account fully for the observed dissociation. Enz et al. [5] showed that HLA-DRB1*15:01 is associated with increased HLA-DRB1/DRB5 expression and greater cortical demyelination measured by MOG loss, an HLA-D-independent pathological readout, yet without a corresponding association with clinical severity. Thus, the dissociation between HLA-related genetics and clinical outcome is not confined to HLA-D-based lesion measures. These observations suggest that HLA genetic burden may influence how a severity-relevant lesion phenotype is represented histologically at end stage, without itself driving the clinical effects of chronic active lesions. This explains why HLA genetic burden correlates with BRL + lesion measures, and BRL + lesions correlate with severity, without HLA genetic burden itself being associated with clinical outcome. Therefore, the apparent dissociation observed in the present study is unlikely to be explained solely by HLA-D-based lesion classification.

Overall, these findings support the conclusion that the adverse clinical profile of cluster 2 is not fully explained by HLA-D-defined chronic active lesion burden in the sampled postmortem tissue. Taken together with the previous findings [5, 12, 13, 16], this observation led us to hypothesize that postmortem lesion burden and genomic clustering reflect two partly independent components of disease severity and that the distribution of HLA genetic burden, and in turn lesion pattern, may partly mask the strong and independent effect of genetic background on disease severity. This interpretation was supported by the finding that adjustment for chronic active lesion burden strengthened, rather than weakened, the association between cluster 2 and worse clinical outcome. If chronic active lesion burden fully explained the enhanced severity of cluster 2, adjustment would be expected to attenuate the cluster effect. Instead, the opposite occurred: after accounting for chronic active lesion burden, cluster 2 showed an even clearer association with ADDS and shorter time from progression to death. This suggests that chronic active lesion burden and genomic cluster assignment capture overlapping but non-identical dimensions of severity. In practical terms, chronic active lesions remain a relevant pathologic marker of aggressive disease, but they do not fully account for the adverse clinical profile associated with cluster 2.

The association of cluster 2 with shorter progression-to-death, but not time-to-progression, further supports the framework proposed by Kreft et al. [15], suggesting that genetically defined MS subtypes diverge mainly in later disease stages, particularly beyond the first decade after disease onset.

These observations, together with the lack of association between HLA-related measures and disease outcomes, support a model in which HLA-linked biology shapes the tissue context in which lesions are classified and interpreted, while genomic clustering and chronic active lesion burden represent partly distinct but converging axes of disease severity. The fact that these axes do not map perfectly onto each other suggests that genomic subtypes may capture components of severity that are only partially visible in terminal lesional and normal-appearing neuropathology. This highlights the need to capture biology beyond lesion features alone when interpreting the pathological correlates of MS severity. More broadly, these findings support the view that no single histological variable is likely to fully explain MS disease severity. Consistent with this view, a recent single-nucleus RNA sequencing study of normal-appearing and lesional white and grey matter tissue in MS found, using multi-omics factor analysis, that differentially expressed genes were more strongly related to patient-level factors than to lesion type [20].

Several limitations should be considered. First, our neuropathological analyses were based on systematically sampled postmortem tissue and therefore reflect end-stage pathology rather than lesion accumulation over time. This may partly explain the discrepancy between the lower chronic active lesion burden observed in cluster 2 at death and the higher T2 lesion accumulation rate reported for the corresponding cluster in the original study. Although the sampled regions covered cortical and non-cortical sites commonly affected in MS, they did not provide whole-brain mapping, and spinal cord pathology was not assessed. We therefore cannot exclude the possibility that cluster-associated pathology is topographically restricted or that unmeasured spinal cord involvement contributed to the greater clinical severity of cluster 2. Thus, the lower frequency of HLA-D-defined chronic active/broad-rim lesions in cluster 2 should not be interpreted as evidence of lower inflammatory activity over time. Second, missing clinical data, missing tissue blocks, and variation in evaluable regions were addressed analytically where possible, but residual sampling effects cannot be excluded. Third, lesion classification relied on HLA-D immunostaining. Because HLA-D expression may itself be influenced by HLA genetic background, HLA-D immunoreactivity cannot be regarded as a genotype-independent measure of inflammation and may influence the histological representation of lesion activity. Alternative markers such as CD68, IBA1, TMEM119/P2RY12, lymphocyte quantification, iron-sensitive measures and axonal injury markers will be required to determine whether the observed differences reflect true inflammatory activity, genetically modulated HLA-D expression, or both. In addition, although MOG immunohistochemistry provides sensitive detection of demyelinated lesions, it may underestimate shadow plaques, partial remyelination, and Wallerian degeneration compared with complementary myelin stains. Finally, although this is a large postmortem MS cohort, smaller subgroup analyses may have been underpowered, which would rather lead to underestimation rather than overestimation of effects. In addition, the study was not powered to identify the specific genes driving the biological effects of genomic clustering and used bulk RNA sequencing, limiting cell-type-specific interpretation. Nevertheless, this study uses a large, deeply phenotyped postmortem cohort with minimal documented exposure to disease-modifying therapies, representative of the wider UK MS Society Tissue Bank holdings. It therefore provides a robust framework for relating genomic stratification to postmortem tissue pathology and supports the central finding that combined susceptibility- and progression-related genetic risk identifies a subgroup of MS patients with a worse prognosis that is not fully explained by end-stage total lesion burden or chronic active lesion burden.

Our findings have several implications for MS biology and biomarker development. First, they suggest that genomic prognostic risk may not map onto neuropathology along a single axis of lesion burden. This is relevant for studies using polygenic risk scores to stratify severity, because genetically defined high-risk groups may not necessarily show concordant increases in conventional lesion-based MRI markers, such as T2 lesion burden or gadolinium-enhancing activity. Severity assessed only through these readouts could therefore miss genetic effects that act through diffuse tissue injury, neurodegeneration, spinal cord involvement, tissue resilience, or other mechanisms not captured by focal inflammatory lesion activity. Second, they indicate that HLA-based histopathologic readouts may be modulated by immunogenetic background, which should be considered when interpreting end-stage tissue phenotypes across genetically defined subgroups. More broadly, these results argue that prognostic genetic stratification should be interpreted alongside, rather than as a surrogate for, lesion-based biomarkers. Future longitudinal imaging and fluid-biomarker studies will be needed to determine which measurable in vivo correlates best capture the adverse biological profile associated with high-risk genomic clusters.

## Supplementary Information

Below is the link to the electronic supplementary material.

Supplementary file1 (PDF 492 KB)

Supplementary file2 (XLSX 69 KB)

## Data Availability

The data and code that support the findings of this study are available from the corresponding author upon reasonable request and subject to approval by the relevant data custodians and governance procedures.

## References

[1] Allen IV, McQuaid S, Mirakhur M, Nevin G (2001) Pathological abnormalities in the normal-appearing white matter in multiple sclerosis. Neurol Sci 22(2):141–144. 10.1007/s100720170012 11603615 10.1007/s100720170012

[2] Bankhead P, Loughrey MB, Fernández JA, Dombrowski Y, McArt DG, Dunne PD et al (2017) QuPath: open source software for digital pathology image analysis. Sci Rep 7(1):16878. 10.1038/s41598-017-17204-5 29203879 10.1038/s41598-017-17204-5 PMC5715110

[3] Cooze B, Neal J, Vineed A, Oliveira JC, Griffiths L, Allen KH et al (2024) Digital pathology identifies associations between tissue inflammatory biomarkers and multiple sclerosis outcomes. Cells 13(12):1020. 10.3390/cells13121020 38920650 10.3390/cells13121020 PMC11201856

[4] Delaneau O, Zagury JF, Marchini J (2013) Improved whole-chromosome phasing for disease and population genetic studies. Nat Methods 10(1):5–6. 10.1038/nmeth.2307 23269371 10.1038/nmeth.2307

[5] Enz LS, Zeis T, Schmid D, Geier F, van der Meer F, Steiner G et al (2019) Increased HLA-DR expression and cortical demyelination in MS links with HLA-DR15. Neurol Neuroimmunol Neuroinflamm 7(2):e656. 10.1212/NXI.0000000000000656 31882398 10.1212/NXI.0000000000000656 PMC6943368

[6] Frischer JM, Weigand SD, Guo Y, Kale N, Parisi JE, Pirko I et al (2015) Clinical and pathological insights into the dynamic nature of the white matter multiple sclerosis plaque. Ann Neurol 78(5):710–721. 10.1002/ana.24497 26239536 10.1002/ana.24497 PMC4623970

[7] Haider L, Zrzavy T, Hametner S, Höftberger R, Bagnato F, Grabner G et al (2016) The topograpy of demyelination and neurodegeneration in the multiple sclerosis brain. Brain 139(Pt 3):807–815. 10.1093/brain/awv398 26912645 10.1093/brain/awv398 PMC4766379

[8] Howell OW, Palser A, Polito A, Melrose S, Zonta B, Scheiermann C et al (2006) Disruption of neurofascin localization reveals early changes preceding demyelination and remyelination in multiple sclerosis. Brain 129(Pt 12):3173–3185. 10.1093/brain/awl290 17041241 10.1093/brain/awl290

[9] Howell OW, Reeves CA, Nicholas R, Carassiti D, Radotra B, Gentleman SM et al (2011) Meningeal inflammation is widespread and linked to cortical pathology in multiple sclerosis. Brain 134(Pt 9):2755–2771. 10.1093/brain/awr182 21840891 10.1093/brain/awr182

[10] Howie B, Fuchsberger C, Stephens M, Marchini J, Abecasis GR (2012) Fast and accurate genotype imputation in genome-wide association studies through pre-phasing. Nat Genet 44:955–959. 10.1038/ng.2354 22820512 10.1038/ng.2354 PMC3696580

[11] International Multiple Sclerosis Genetics Consortium (2019) Multiple sclerosis genomic map implicates peripheral immune cells and microglia in susceptibility. Science 365(6460):eaav7188. 10.1126/science.aav7188 31604244 10.1126/science.aav7188 PMC7241648

[12] International Multiple Sclerosis Genetics Consortium, MultipleMS Consortium (2023) Locus for severity implicates CNS resilience in progression of multiple sclerosis. Nature 619(7969):323–331. 10.1038/s41586-023-06250-x 37380766 10.1038/s41586-023-06250-x PMC10602210

[13] Jokubaitis VG, Campagna MP, Ibrahim O, Stankovich J, Kleinova P, Matesanz F et al (2023) Not all roads lead to the immune system: the genetic basis of multiple sclerosis severity. Brain 146(6):2316–2331. 10.1093/brain/awac449 36448302 10.1093/brain/awac449

[14] Klotz L, Smolders J, Lehto J, Matilainen M, Lütje L, Buchholz L et al (2025) Broad rim lesions are a new pathological and imaging biomarker for rapid disease progression in multiple sclerosis. Nat Med 31(6):2016–2026. 10.1038/s41591-025-03625-7 40301560 10.1038/s41591-025-03625-7 PMC12176629

[15] Kreft KL, Mekkes NJ, Uzochukwu E, Loveless S, Wynford-Thomas R, Harding KE et al (2026) Genetic subtypes associated with multiple sclerosis severity and response to treatment. J Neurol Neurosurg Psychiatry 97(5):413–421. 10.1136/jnnp-2025-337337 41545194 10.1136/jnnp-2025-337337

[16] Kreft KL, Uzochukwu E, Loveless S, Willis M, Wynford-Thomas R, Harding KE et al (2024) Relevance of multiple sclerosis severity genotype in predicting disease course: a real-world cohort. Ann Neurol 95(3):459–470. 10.1002/ana.26831 37974536 10.1002/ana.26831

[17] Kutzelnigg A, Lucchinetti CF, Stadelmann C, Brück W, Rauschka H, Bergmann M et al (2005) Cortical demyelination and diffuse white matter injury in multiple sclerosis. Brain 128(Pt 11):2705–2712. 10.1093/brain/awh641 16230320 10.1093/brain/awh641

[18] Langer-Gould A, Popat RA, Huang SM, Cobb K, Fontoura P, Gould MK et al (2006) Clinical and demographic predictors of long-term disability in patients with relapsing-remitting multiple sclerosis: a systematic review. Arch Neurol 63(12):1686–1691. 10.1001/archneur.63.12.1686 17172607 10.1001/archneur.63.12.1686

[19] Luchetti S, Fransen NL, van Eden CG, Ramaglia V, Mason M, Huitinga I (2018) Progressive multiple sclerosis patients show substantial lesion activity that correlates with clinical disease severity and sex: a retrospective autopsy cohort analysis. Acta Neuropathol 135(4):511–528. 10.1007/s00401-018-1818-y 29441412 10.1007/s00401-018-1818-y PMC5978927

[20] Macnair W, Calini D, Agirre E, Bryois J, Jäkel S, Smith RS et al (2025) snRNA-seq stratifies multiple sclerosis patients into distinct white matter glial responses. Neuron 113(3):396-410.e9. 10.1016/j.neuron.2024.11.016 39708806 10.1016/j.neuron.2024.11.016

[21] Magliozzi R, Howell O, Vora A, Serafini B, Nicholas R, Puopolo M et al (2007) Meningeal B-cell follicles in secondary progressive multiple sclerosis associate with early onset of disease and severe cortical pathology. Brain 130(Pt 4):1089–1104. 10.1093/brain/awm038 17438020 10.1093/brain/awm038

[22] Martin JE, Raffel J, Nicholas R (2016) Progressive dwindling in multiple sclerosis: an opportunity to improve care. PLoS ONE 11(7):e0159210. 10.1371/journal.pone.0159210 27441557 10.1371/journal.pone.0159210 PMC4956073

[23] Nicholas R, Magliozzi R, Marastoni D, Howell O, Roncaroli F, Muraro P et al (2024) High levels of perivascular inflammation and active demyelinating lesions at time of death associated with rapidly progressive multiple sclerosis disease course: a retrospective postmortem cohort study. Ann Neurol 95(4):706–719. 10.1002/ana.26870 38149648 10.1002/ana.26870

[24] Reich DS, Lucchinetti CF, Calabresi PA (2018) Multiple sclerosis. N Engl J Med 378(2):169–180. 10.1056/NEJMra1401483 29320652 10.1056/NEJMra1401483 PMC6942519

[25] Sartoris S, Del Pozzo G (2024) Exploring the HLA complex in autoimmunity: from the risk haplotypes to the modulation of expression. Clin Immunol 265:110266. 10.1016/j.clim.2024.110266 38851519 10.1016/j.clim.2024.110266

