# Supplementary material for: Genetic subtypes of multiple sclerosis severity are uncoupled from inflammatory lesion burden
Source: Acta Neuropathol. 2026 Aug 21;152(1):24. doi: 10.1007/s00401-026-03062-x (PMC13498620; doi:10.1007/s00401-026-03062-x)
Supplement: Supplementary file 1 [file 401_2026_3062_MOESM1_ESM.pdf]

Supplementary Figures

Title: Genetic subtypes of multiple sclerosis severity are uncoupled from inflammatory lesion burden

Authors: Gianmarco Abbadessa, Karim L. Kreft, Owain Howell, Jean Victor Carlos de Oliveira, Benjamin Cooze, Artemis Papadaki, Joic Majo Jacinto, Ildiko Farkas, Yeung-Yeung Leung, Simona Bonavita, Emma Clare Tallantyre, Neil P. Robertson, Ai Nagano, Mie Rizig, Roberta Magliozzi, David Owen, Richard Reynolds, Richard Nicholas

Corresponding authors: Richard Nicholas and Karim L. Kreft

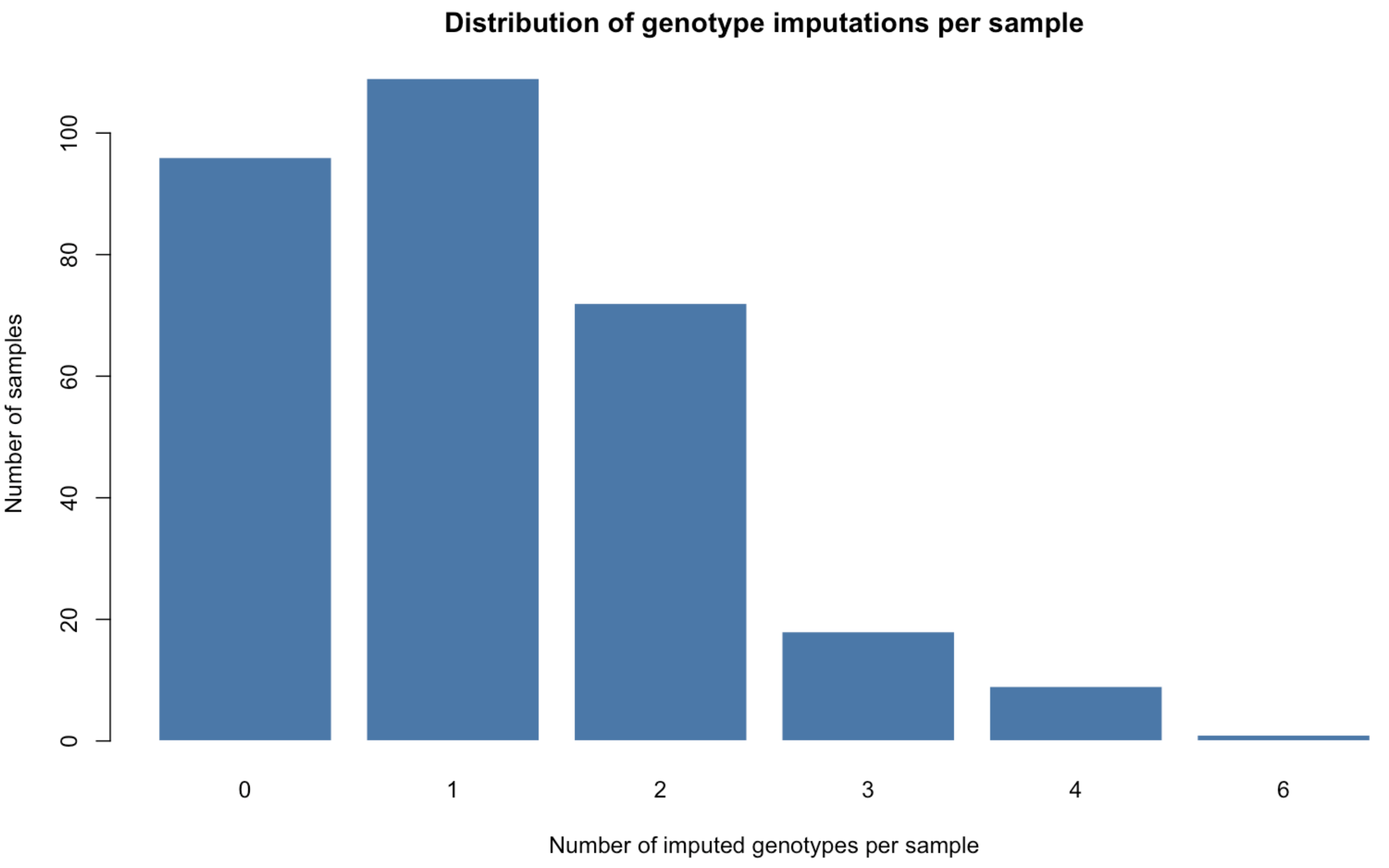

Figure S1. Distribution of imputed genotypes per sample.

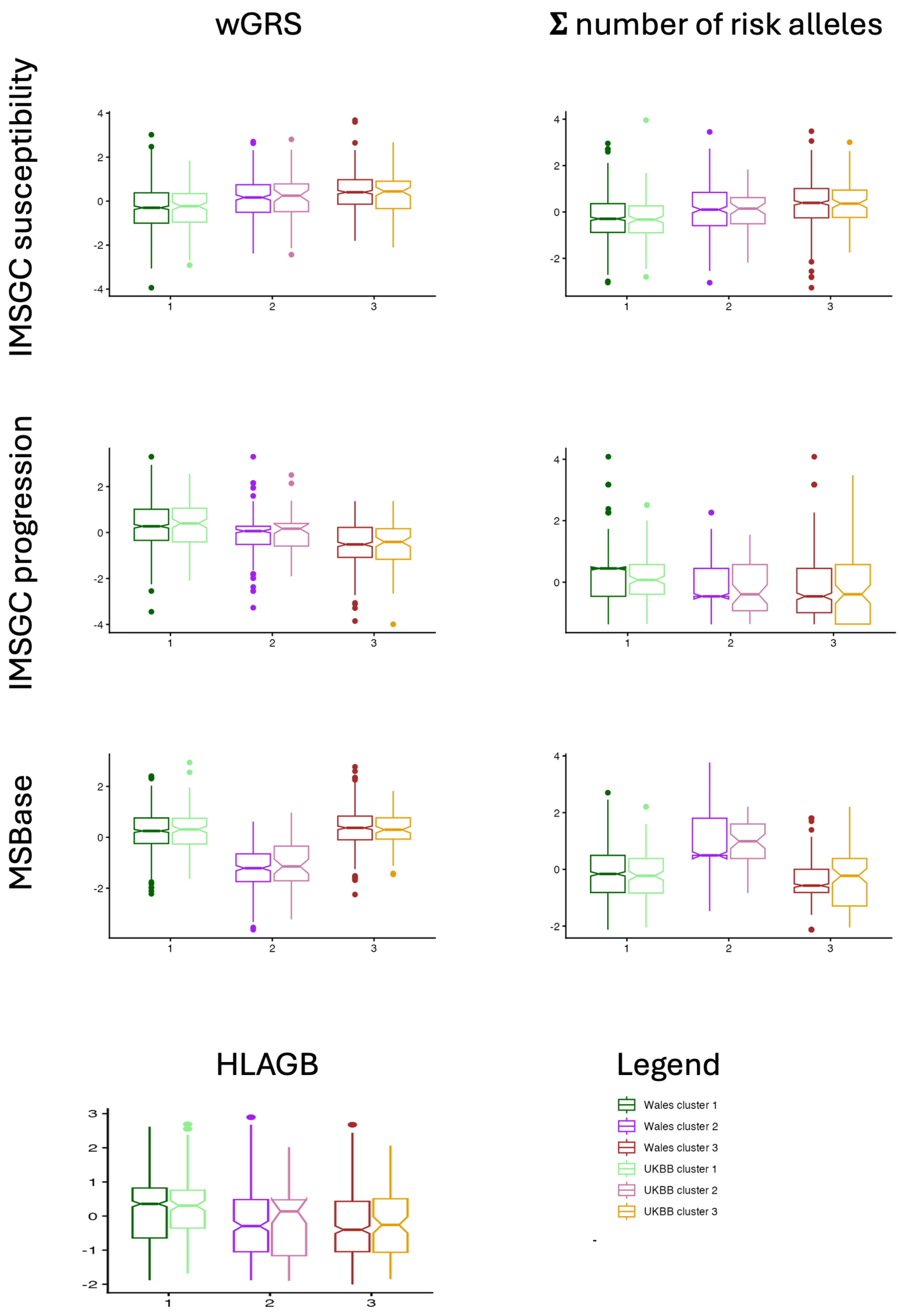

Figure S2. Comparison of genetic scores across genomic clusters between the Wales and the MS UK Tissue bank populations.

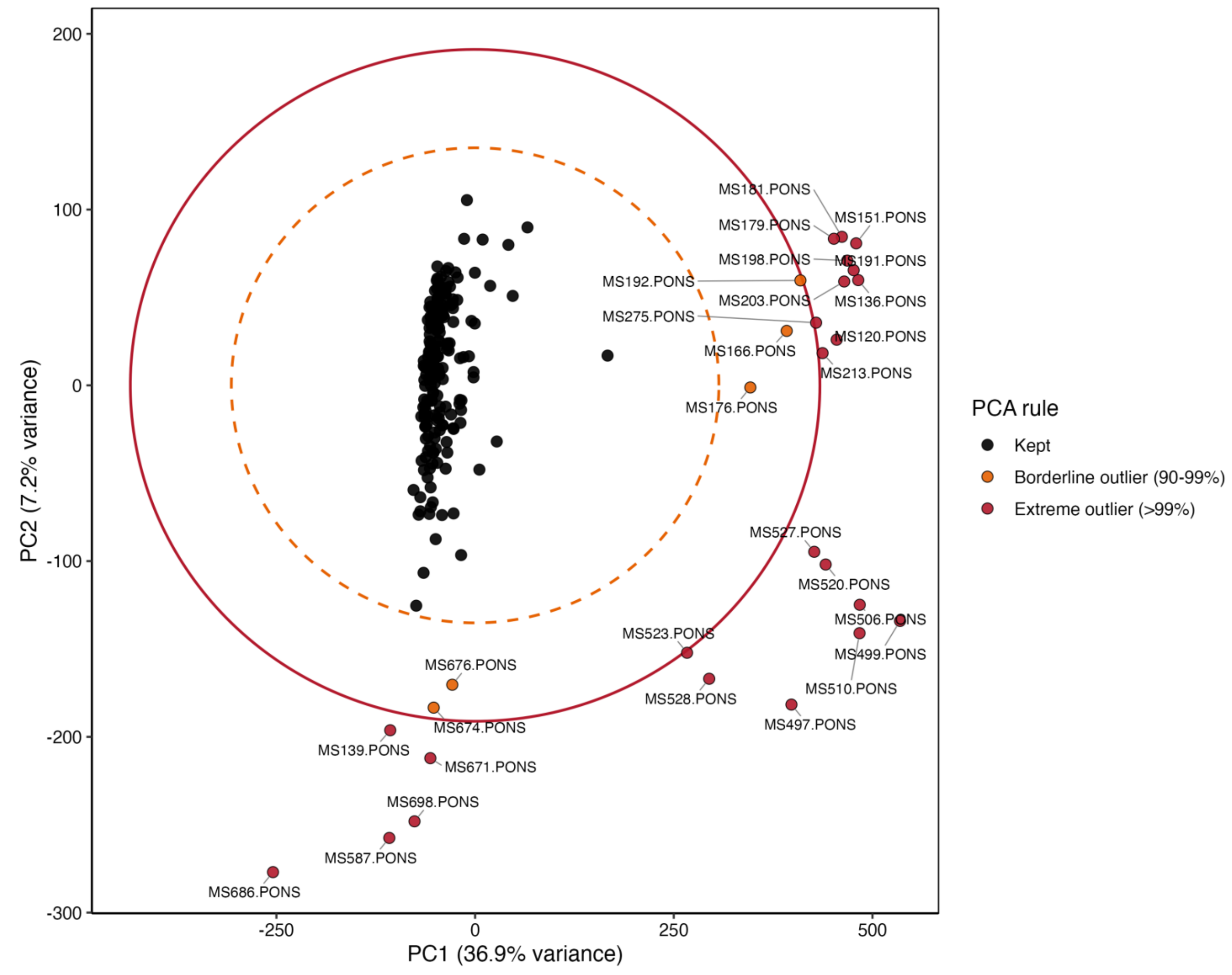

Figure S3. Principal component analysis of the pons RNA sequencing dataset used for outlier detection. Samples outside the 99% Mahalanobis-distance threshold were classified as extreme outliers, and samples between the 90% and 99% thresholds were classified as borderline outliers; both groups were excluded from downstream expression analyses.
